# Supplementary material for: Machine learning-based tsunami inundation prediction derived from offshore observations
Source: Nat Commun. 2022 Sep 19;13:5489. doi: 10.1038/s41467-022-33253-5 (PMC9485236; doi:10.1038/s41467-022-33253-5)
Supplement: Supplementary file 1 — Supplementary Information [file 41467_2022_33253_MOESM1_ESM.pdf]

Supplementary Information for

Machine learning-based tsunami inundation prediction derived from offshore observations

Iyan E. Mulia[1, 2*], Naonori Ueda[1, 2], Takemasa Miyoshi[1, 3], Aditya Riadi Gusman[4], Kenji Satake[5]

[1]Prediction Science Laboratory, RIKEN Cluster for Pioneering Research, Kobe, Japan.
[2]Disaster Resilience Science Team, RIKEN Center for Advanced Intelligence Project, Tokyo, Japan.
[3]Data Assimilation Research Team, RIKEN Center for Computational Science, Kobe, Japan.
[4]GNS Science, Lower Hutt, New Zealand.
[5]Earthquake Research Institute, The University of Tokyo, Tokyo, Japan.

[*]iyan.mulia@riken.jp

Supplementary information including:

Supplementary Fig. 1.
Supplementary Fig. 2.
Supplementary Fig. 3.
Supplementary Fig. 4.
Supplementary Fig. 5.
Supplementary Fig. 6.
Supplementary Fig. 7.
Supplementary Fig. 8.
Supplementary Fig. 9.
Supplementary Fig. 10.
Supplementary Fig. 11.
Supplementary Fig. 12.

Supplementary Table 1.

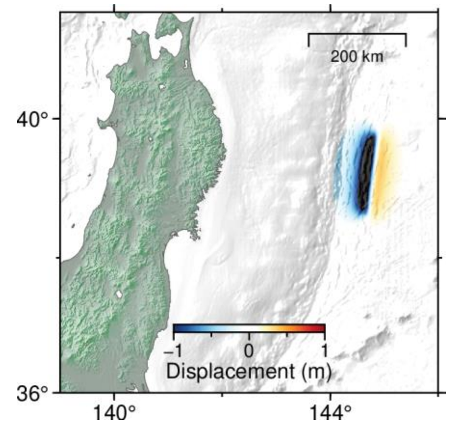

Supplementary Fig. 1. An example of tsunami source (vertical displacement) from an outer-rise earthquake of magnitude *M*w 7.9

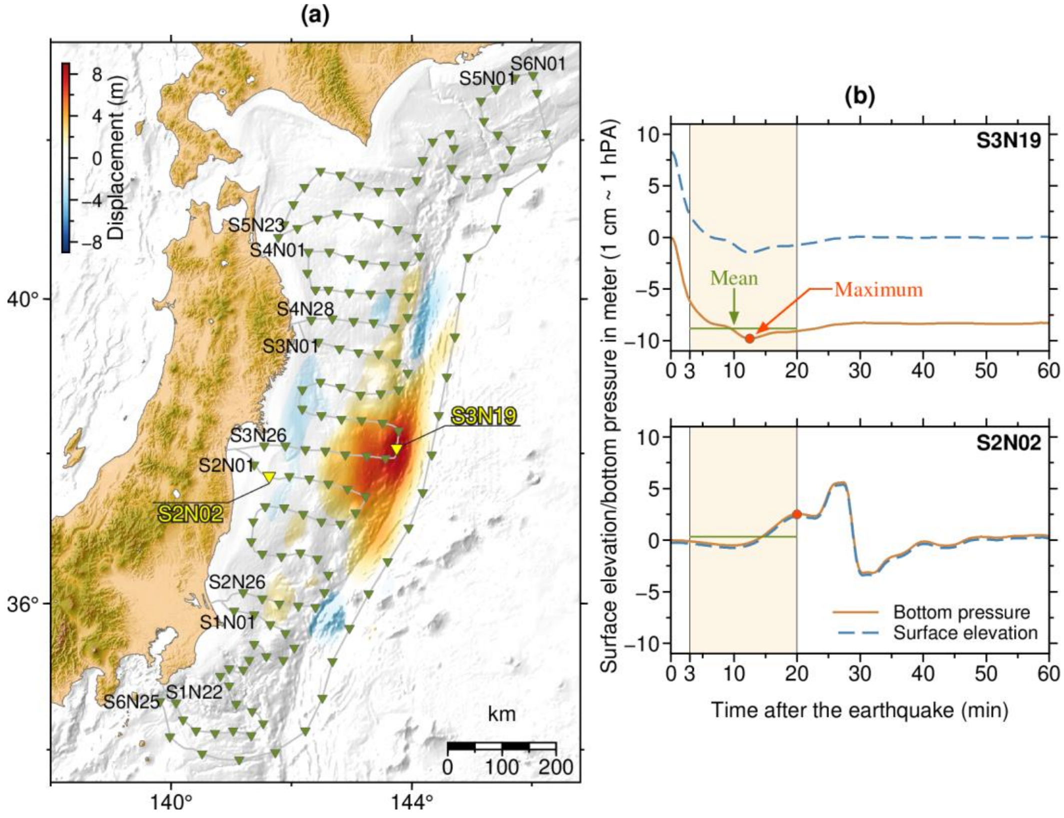

Supplementary Fig. 2. a S-net stations and a source model or vertical displacement of the 2011 Tohoku-oki event. Station names at both ends of each segment and selected stations are annotated. **b** Tsunami amplitudes from the source model in terms of surface elevation and bottom pressure at stations marked in **a**. Mean and maximum tsunami amplitudes used as model inputs are annotated. The shaded area indicates the prediction window of 20 min as an example..

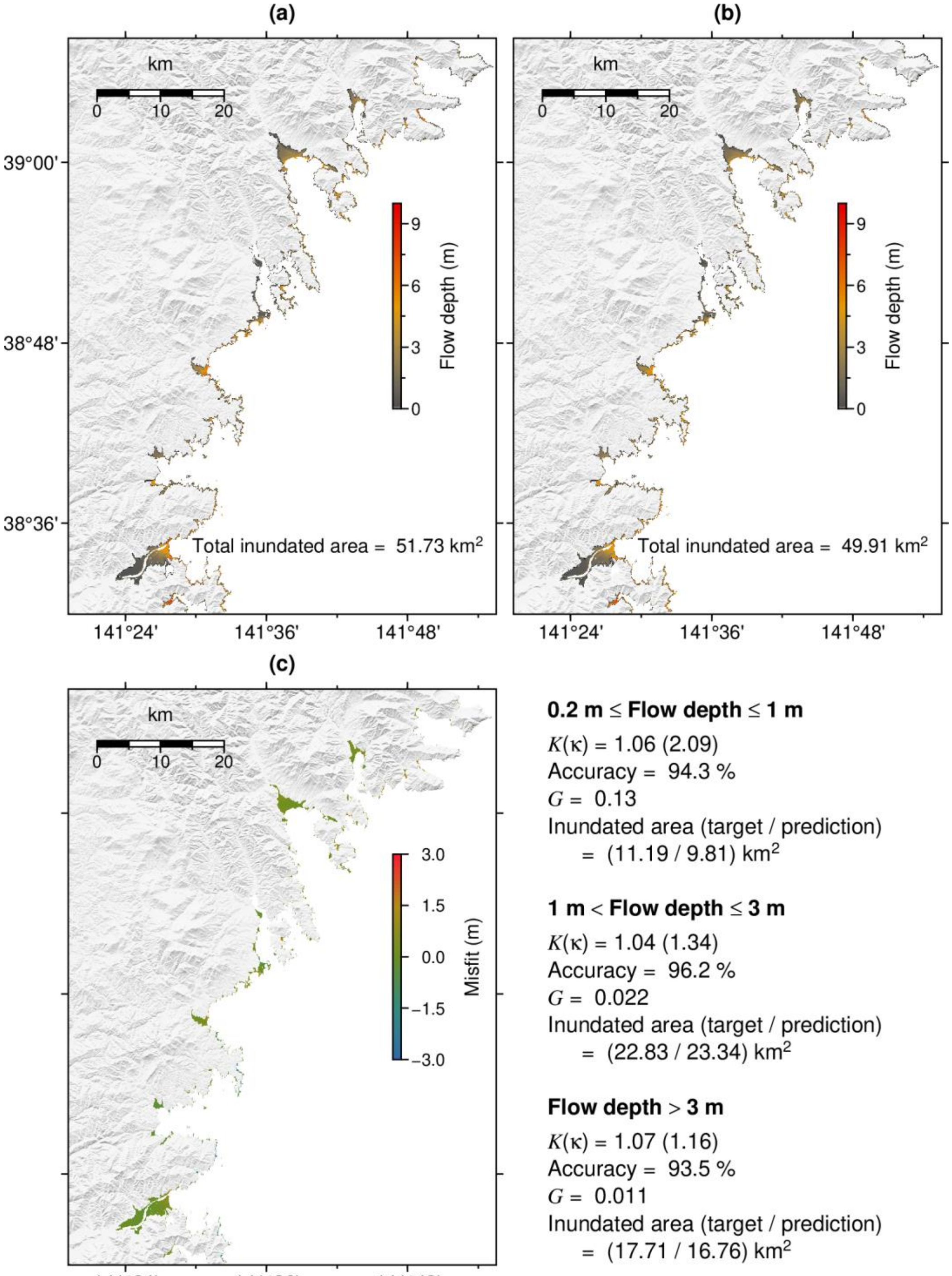

Supplementary Fig. 3. a A sample of target inundation map on the test set for an earthquake magnitude of *M*w 8.2. **b** Predicted inundation map using the 20-min window. **c** The map of misfit and statistical evaluation results.

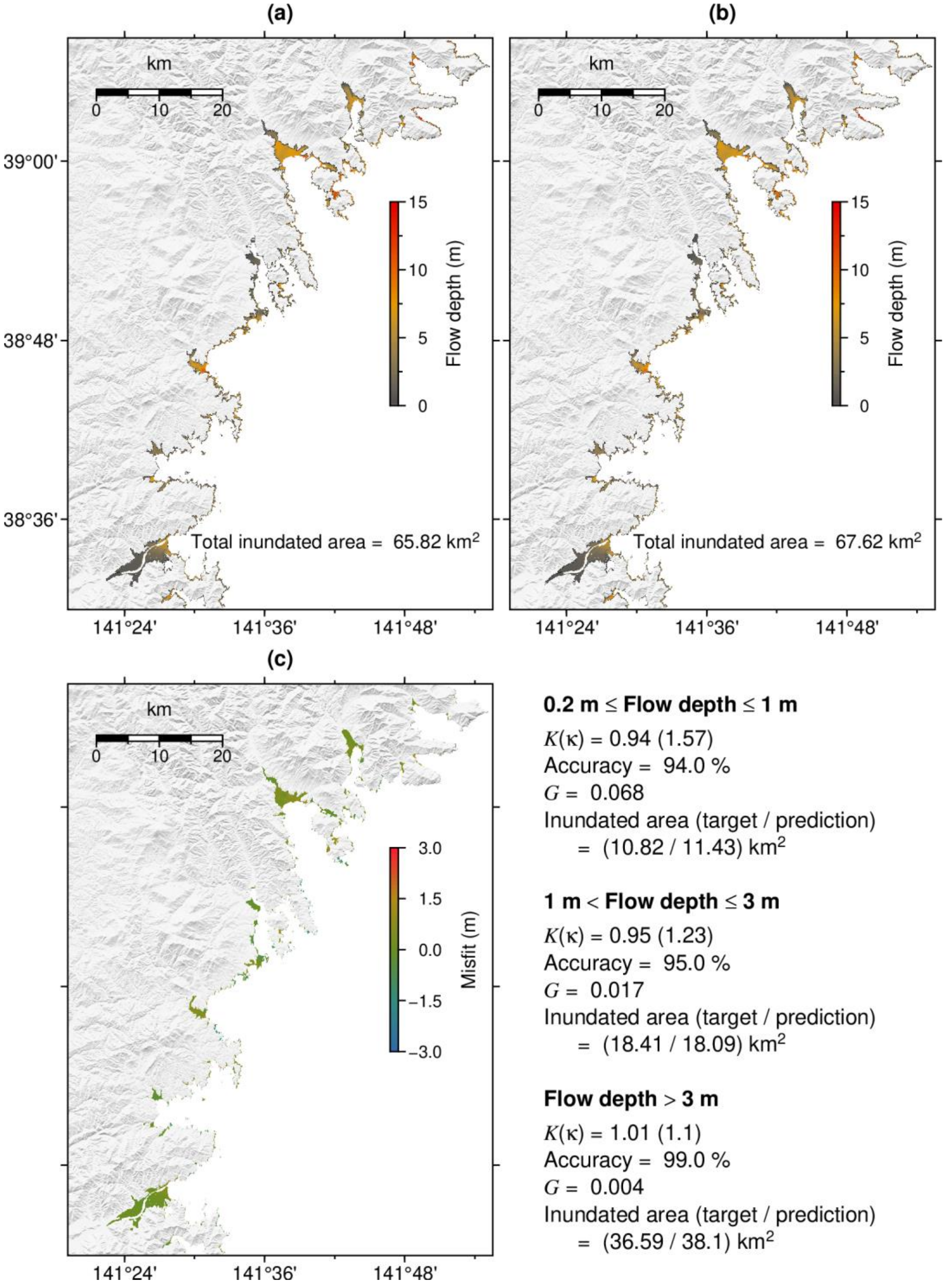

Supplementary Fig. 4. Same with Supplementary Fig. 3 for *M*w 8.5

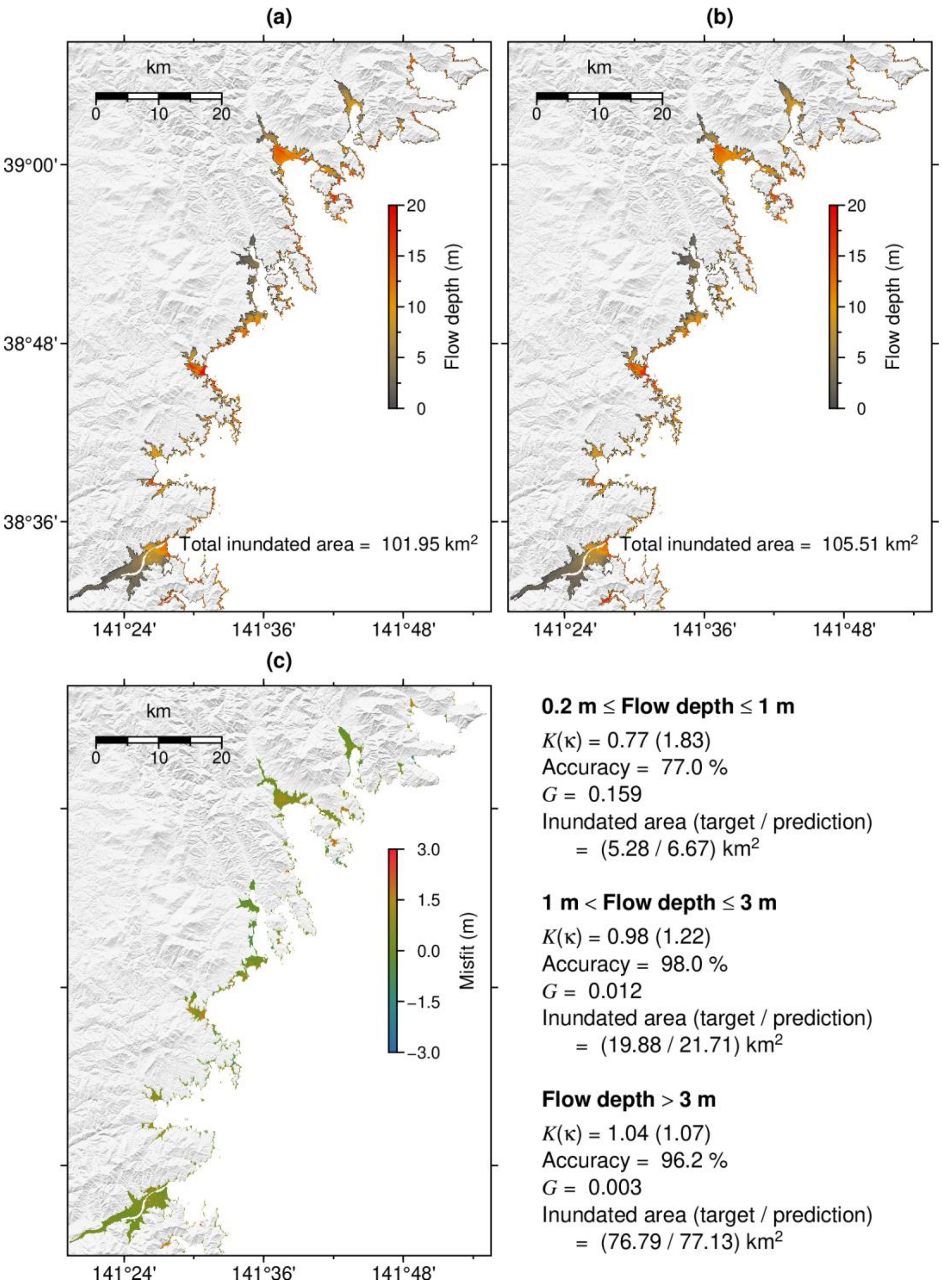

Supplementary Fig. 5. Same with Supplementary Fig. 3 for *M*w 8.8.

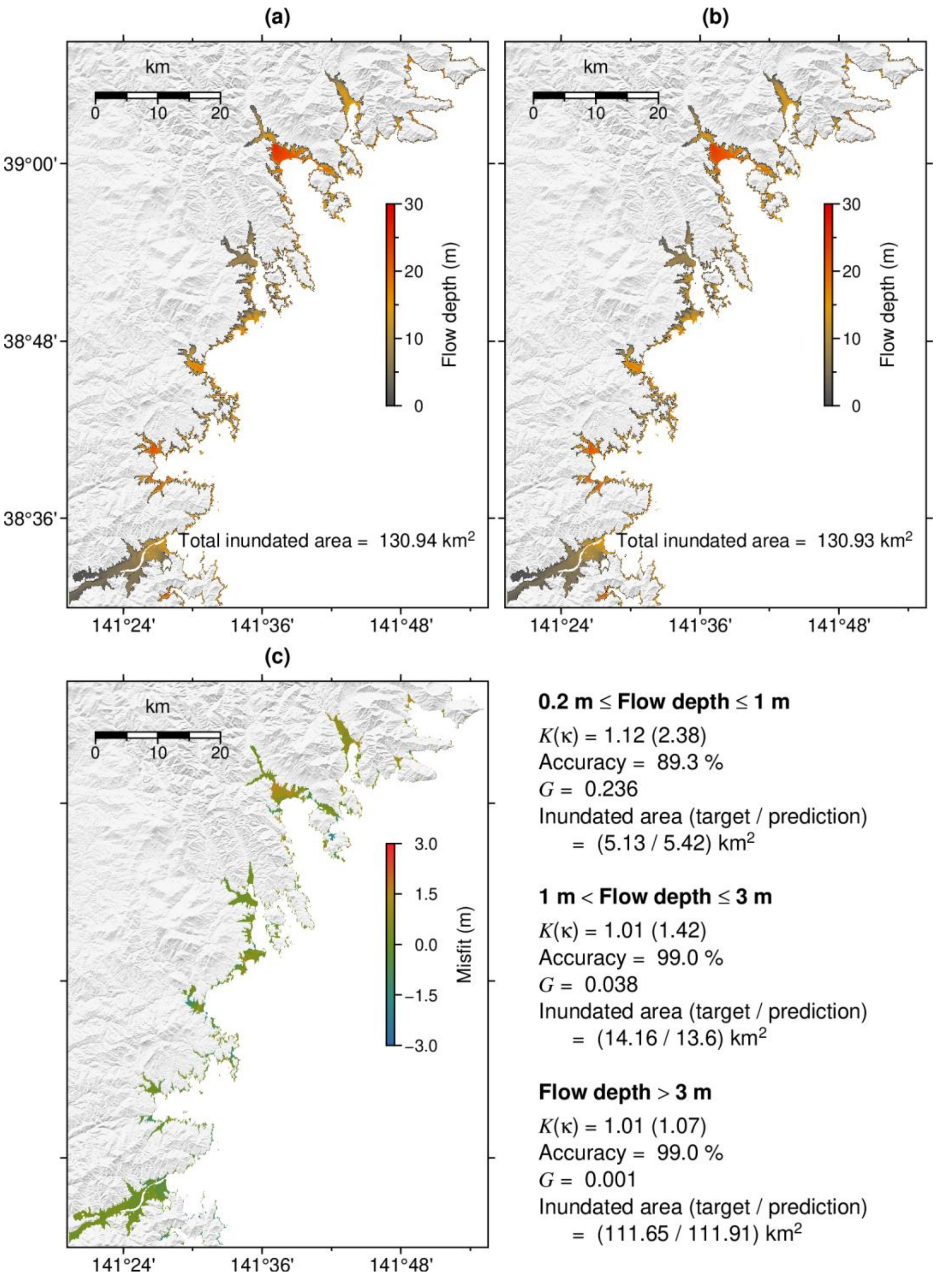

Supplementary Fig. 6. Same with Supplementary Fig. 3 for *M*w 9.1.

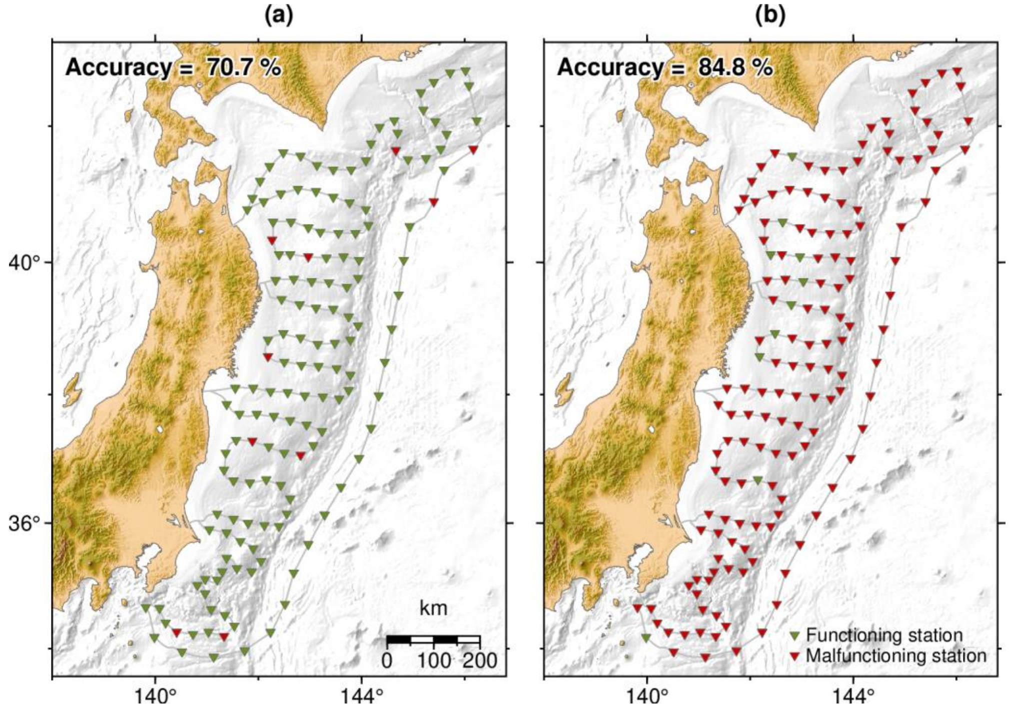

Supplementary Fig. 7. a A combination of 10 malfunctioning stations resulting in the lowest accuracy. **b** A combination of 140 malfunctioning stations resulting in the highest accuracy.

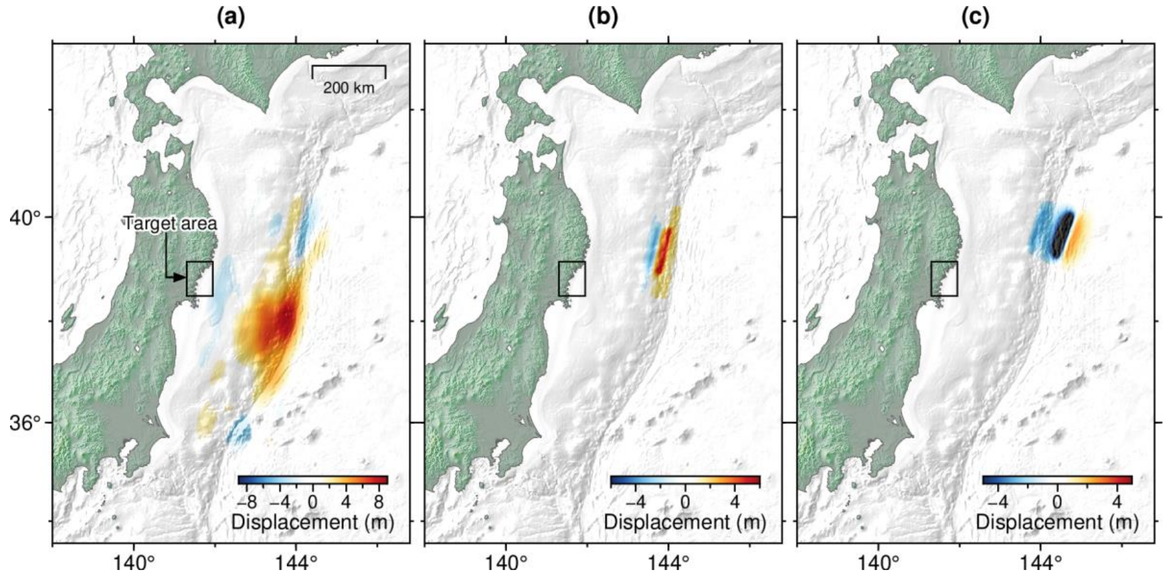

Supplementary Fig. 8. Tsunami source models of the 2011 Tohoku-oki (**a**), the 1896 Meiji Sanriku (**b**), and the 1933 Showa Sanriku (**c**) events.

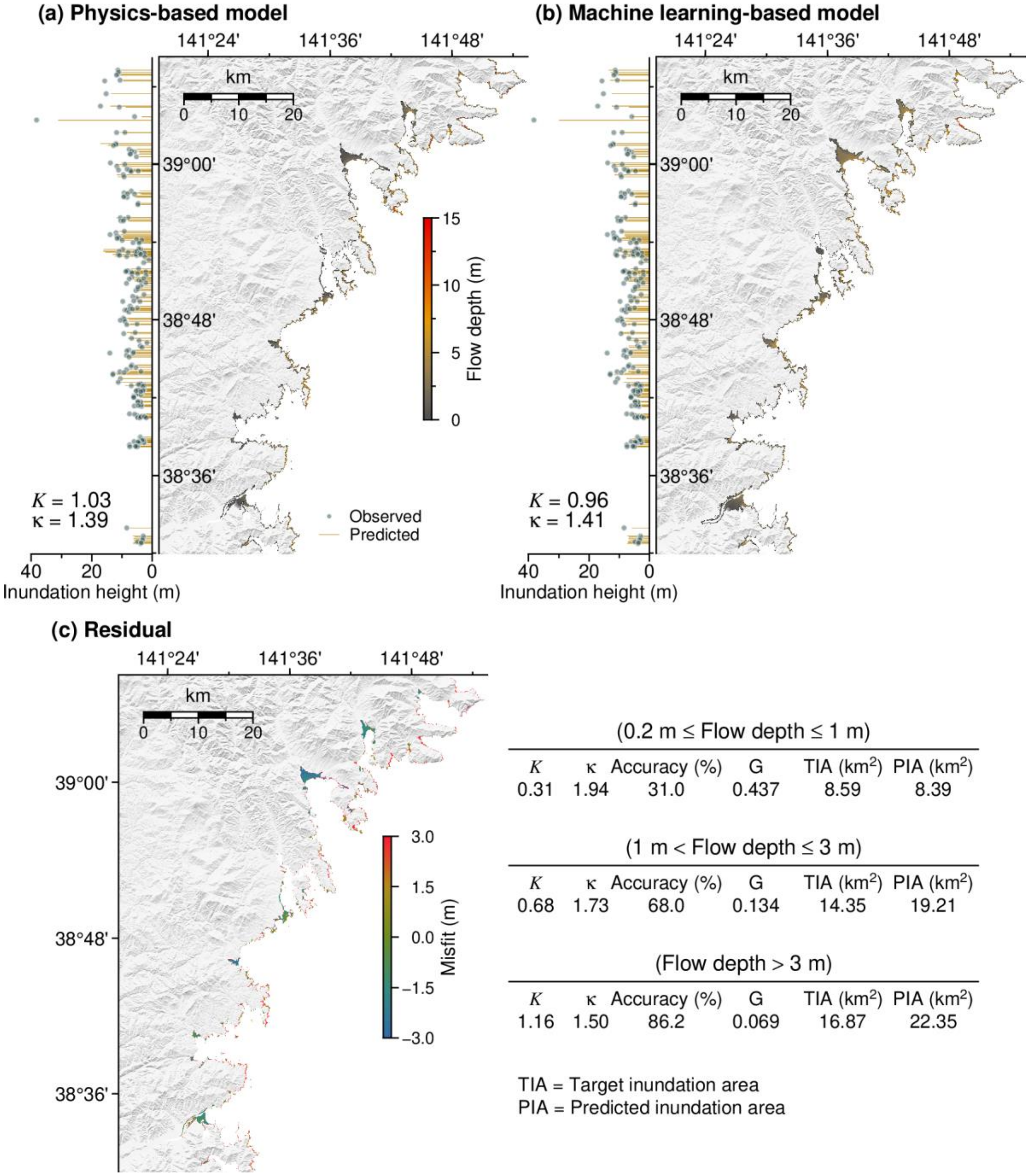

Supplementary Fig. 9. Inundation map in terms of flow depths at locations of interest (right panel) and comparisons between observed and predicted inundation heights (left panel) for the 1896 Meiji Sanriku event. **a** Physics-based model. **b** Machine learning-based model. **c** The misfit [**a** - **b**] and statistical evaluations of **b** relative to **a**.

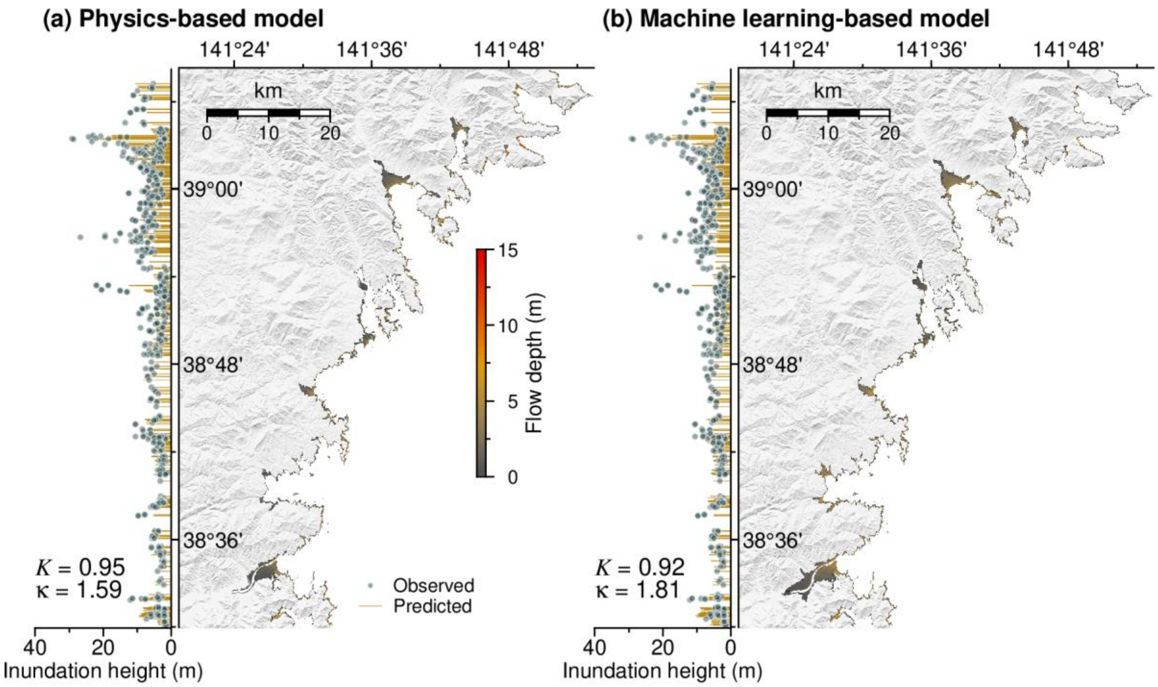

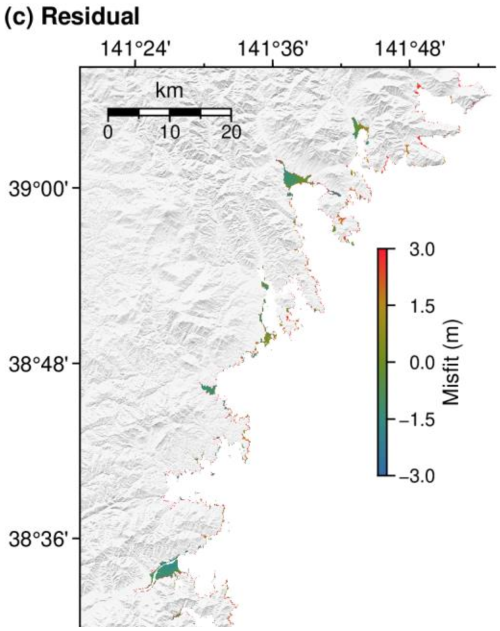

(0.2 m ≤ Flow depth ≤ 1 m)

K	κ	Accuracy (%)	G	TIA (km^2)	PIA (km^2)
0.38	2.14	38.0	0.393	9.90	10.42

(1 m < Flow depth ≤ 3 m)

K	κ	Accuracy (%)	G	TIA (km^2)	PIA (km^2)
0.76	1.74	76.0	0.099	20.13	24.10

(Flow depth > 3 m)

K	κ	Accuracy (%)	G	TIA (km^2)	PIA (km^2)
1.22	1.53	82.0	0.130	14.10	18.53

TIA = Target inundation area
PIA = Predicted inundation area

Supplementary Fig. 10. Inundation map in terms of flow depths at locations of interest (right panel) and comparisons between observed and predicted inundation heights (left panel) for the 1933 Showa Sanriku event. **a** Physics-based model. **b** Machine learning-based model. **c** The misfit [**a** - **b**] and statistical evaluations of **b** relative to **a**.

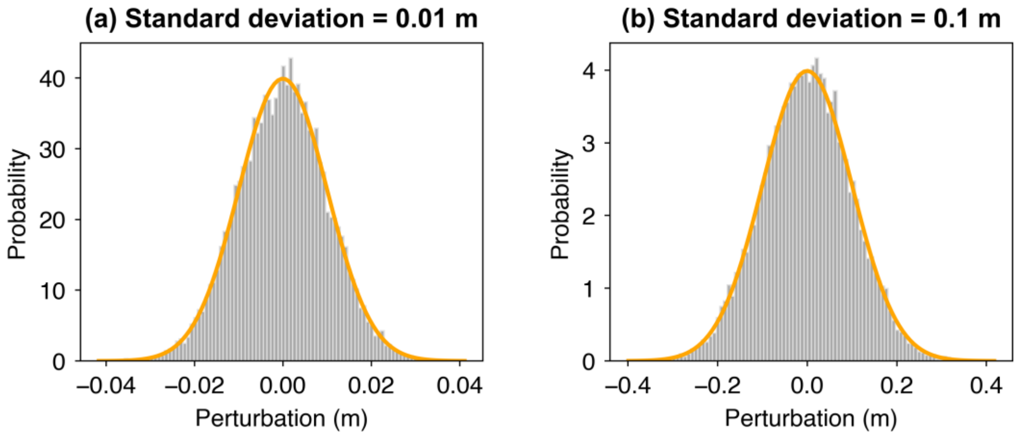

Supplementary Fig. 11. a The probability density function of perturbations with a standard deviation of 0.01 m. **b** The probability density function of perturbations with a standard deviation of 0.1 m. Orange lines indicate normal probability density functions.

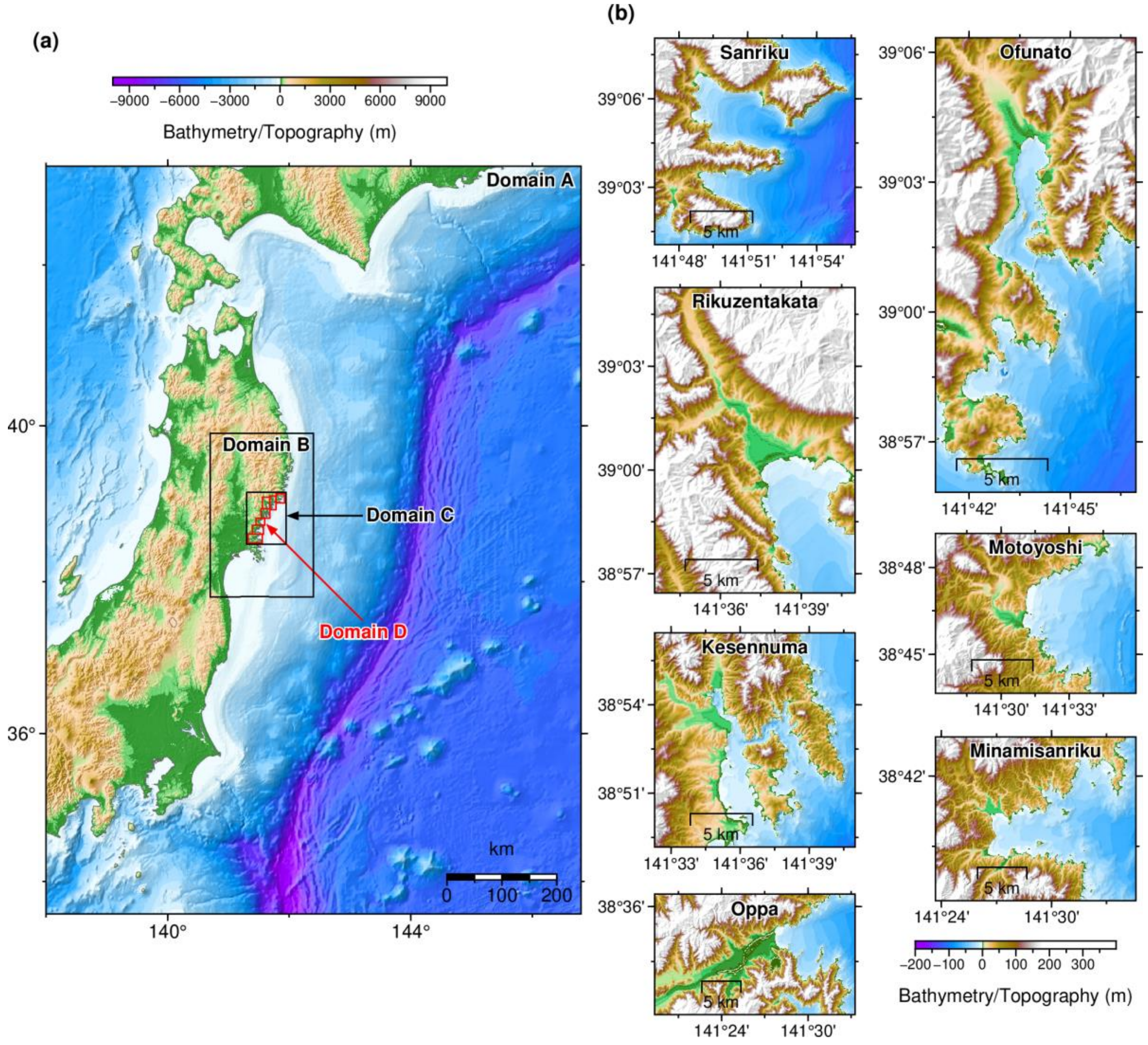

Supplementary Fig. 12. a Nested grid domains for the physics-based tsunami simulation and bathymetry and topography of the largest domain. **b** Bathymetry and topography around the coastal cities of Sanriku, Ofunato, Rikuzentakata, Motoyoshi, Kesennuma, Minamisanriku and Oppa in the smallest modeling domains.

Supplementary Table 1. Medians and 25th and 75th percentiles of the goodness-of-fit statistic for each magnitude corresponded to Fig 4c in the main text.

***M*w**	**Median**	**25th percentile**	**75th percentile**
8.0	0.076	0.054	0.136
8.1	0.086	0.039	0.142
8.2	0.066	0.029	0.114
8.3	0.060	0.013	0.146
8.4	0.033	0.015	0.074
8.5	0.021	0.009	0.066
8.6	0.021	0.009	0.045
8.7	0.020	0.007	0.034
8.8	0.013	0.003	0.028
8.9	0.004	0.002	0.009
9.0	0.007	0.003	0.014
9.1	0.004	0.003	0.007
